# The role of mast cell tryptases in cardiac myxoma: Histogenesis and development of a challenging tumor

**DOI:** 10.3892/ol.2014.2104

**Published:** 2014-04-29

**Authors:** GIUSEPPE DONATO, FRANCESCO CONFORTI, CATERINA CAMASTRA, MICHELE AMMENDOLA, ANNALIDIA DONATO, ATTILIO RENZULLI

**Affiliations:** 1 Department of Pathology, School of Medicine, University Magna Graecia, Catanzaro I-88100, Italy; 2 Department of Pharmacology, School of Medicine, University Magna Graecia, Catanzaro I-88100, Italy; 3 Department of Cardiac Surgery, School of Medicine, University Magna Graecia, Catanzaro I-88100, Italy

**Keywords:** cardiac myxoma, mast cells, tryptase, angiogenesis

## Abstract

A number of available studies have focused on the role of mastocytes and their angiogenic factors, such as tryptase expression, in cancer growth as a major research objective. Cardiac myxoma is a rare neoplasia and is the most common primary tumor of the heart. The cellular elements of cardiac myxoma have an endothelial phenotype; however, its histogenesis remains unclear. Currently, no available studies have correlated the pathological characteristics of cardiac myxomas, such as cell differentiation and vascularization, with the angiogenic factors of mast cells. The aim of the present study was to investigate the role of mast cell tryptases on the development of cardiac myxomas and examine the histogenesis of tumoral cells. A series of 10 cardiac myxomas were examined by immunohistochemical analysis for the presence of tryptase-positive mast cells. Statistical analysis of our data demonstrated that angiogenesis and the development of pseudovascular structures were correlated with the number of tryptase-positive mast cells. Therefore, we hypothesize that cardiac myxoma cells are endothelial precursors which are able to generate mature vascular structures. Further morphological and immunophenotypic analyses of tumoral cells may corroborate such a hypothesis.

## Introduction

Primary cardiac tumors are rare, with an autopsy incidence ranging from 0.001 to 0.03% (1). Cardiac myxoma is the most common primary cardiac tumor worldwide, and myxomas may be sporadic or part of genetic conditions, such as Carney complex or lentigines, atrial myxoma and blue nevi syndrome (2,3).

Microscopically, myxomas exhibit a myxoid stroma with plump spindle or stellate cells. Such elements have endothelial characteristics and may be organized into pseudovascular structures. In certain cases, a variably abundant vascular component may also be present (4).

An increase in the number of mast cells in tumors and a correlation between angiogenesis, mast cell number and growth of the neoplasm has previously been reported (5–7).

Currently, no available studies have demonstrated that the pathological characteristics of cardiac myxomas, such as cell differentiation and vascularization, are correlated with the angiogenic factors of mast cells. In the present study, via immunohistochemical analysis, the role of mast cell tryptases in cardiac myxomas was investigated using a series of 10 cardiac myxomas (8). Furthermore, the possible association between the tumorigenesis of myxomas and current theories regarding endocardial development were investigated.

## Materials and methods

### Materials

Archival formalin-fixed and paraffin-embedded tissues were used to study sporadic left atrial myxomas and were collected from 10 consecutive patients (four male and six female patients; mean age, 56±4.7 years) who had undergone surgery at the Department of Cardiac Surgery, School of Medicine, University Magna Graecia (Catanzaro, Italy). The study was approved by the ethics committee of the University Magna Graecia (Catanzaro, Italy). Serial deparaffinated sections (4 μm-thick) were used for the staining procedures, including hematoxylin and eosin, Alcian Blue (pH 2.5; Bio-Optica Milano SpA, Milano, Italy) and immunohistochemistry. All the procedures were performed at room temperature. Patients provided written informed consent.

### Immunohistochemistry

Mast cells in all cases were then immunohistochemically stained for mouse monoclonal anti-human tryptase (clone 10D11, 1:150 dilution; Leica, Mannheim, Germany), mouse monoclonal anti-human cluster of differentiation (CD)31 (clone JC70A, 1:40 dilution; Dako, Carpinteria, CA, USA), mouse monoclonal anti-human CD34 (clone QBEnd10, 1:250 dilution; Dako) and rabbit polyclonal anti-human CD117 (1:100 dilution; Dako) with an automated immunostainer (Bond™ Max; Leica Biosystems, Melbourne, Australia) (9).

Blood vessel density was assessed by light microscopy according to the method of Weidner *et al* (10) and a score graded on a scale of one to four was assigned: 1, 1–5 microvessels observed; 2, 6–10 microvessels observed; 3, 11–15 microvessels observed; 4, 16–20 microvessels observed.

### Evaluation of positive cells

**Figure 1:**
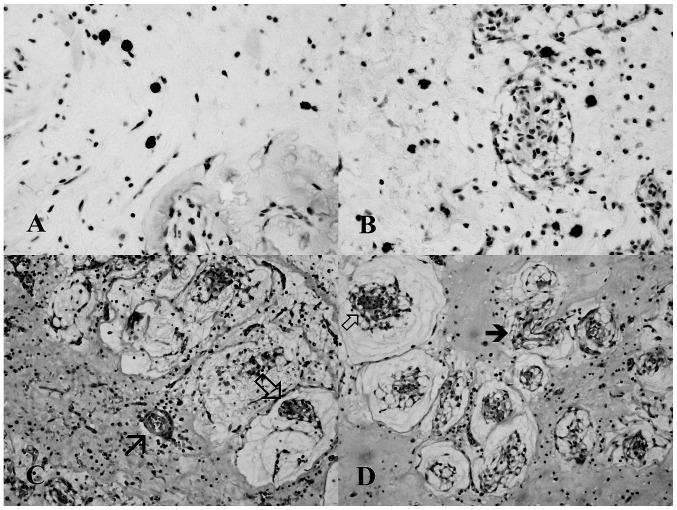
Scattered, dark stained, cellular elements were observed in the immunohistochemical staining of mast cells for (A) tryptase and (B) cluster of differentiation 117. Hematoxylin and eosin staining revealed (C) pseudovascular structures without intermixed vessels (empty arrow) and an isolated vessel-like channel (full arrow), and (D) pseudovascular (full arrow) and vascular structures growing in the same area, and vascular channels developing in the pseudovascular structures (empty arrow). Scale bar, A–D=100 μm.

The number of mast cells that were tryptase-positive (Fig. 1A) and CD117-positive cells (Fig. 1B) was evaluated according to the method of Benitez-Bribiesca *et al* (7).

### Statistical analysis

Statistical analysis was performed in order to calculate the correlation coefficient using least square regression analysis between the blood vessel density score and the number of tryptase-positive mast cells, as well as the associated P-value.

Histopathological characteristics, such as the presence of pseudovascular structures, abundant (≥10 pseudovascular channels in five high power fields; magnification, ×400) or scanty (<10 pseudovascular channels in five high power fields; magnification, ×400, and the presence or absence of hemorrhages were recorded. Pseudovascular structures were recognized as the lumen lacked red blood cells and was lined by larger, often multinucleated, cells.

Student’s t-test was used to compare the number of tryptase-positive mast cells in the two groups of tumors with abundant or scanty pseudovascular structures. In addition, the correlation between the tumor size and the number of tryptase-positive cells was examined by a correlation index. P<0.05 was considered to indicate a statistically significant difference. Analyses were performed using the online ‘Statistics to Use’ software (http://www.physics.csbsju.edu/stats/).

## Results

### Main findings

The immunohistochemical findings and tumor size are summarized in Tables I and II, respectively. Statistical analysis demonstrated a positive correlation between angiogenesis and the number of tryptase-positive mast cells (r=0.797; P=0.006).

### Results of the statistical analysis

The results of Student’s *t*-test allowed us to reject the null hypothesis in our series (P=0.009) concerning the two groups of tumors with abundant and scanty pseudovascular structures. Moreover, the number of CD117-positive cells, attributed only to mast cells, as basophils, endothelial and neoplastic elements are known to be only feebly positive or negatively stained (11,12), were increased in all the cases compared with the number of tryptase-positive elements, suggesting degranulation of mastocytes (Table I). Tumor size was not correlated with the number of tryptase-positive cells (r=0.584; P=0.076).

### Morphological observations

Qualitative analysis of our series suggests that developing pseudovascular structures may be segregated or intermixed with vessels. Notably, such structures clearly originate from these complex architectures (Fig. 1C and D).

**Figure 2:**
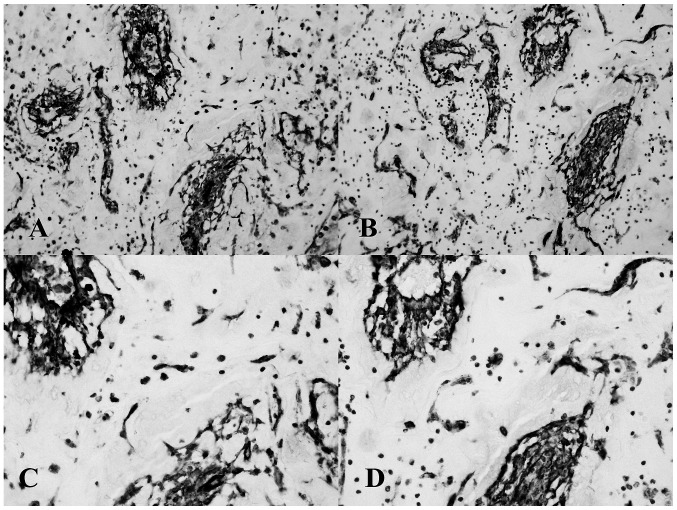
In the same area of a myxoma, pseudovascular structures and scattered tumoral cells stained positive for (A) CD31, (B) CD34 (magnification, ×20), (C) CD31 and (D) CD34 (magnification, ×40). Scale bars, A and B=100 μm; C and D=200 μm. CD, cluster of differentiation.

Isolated tumor tissues were CD31- and CD34-positive with an irregular staining distribution on the cell membrane; furthermore, tumor tissues exhibiting vascular and pseudovascular structures were always stained positive for CD31 and CD34 (Fig. 2A and D).

## Discussion

Cardiac myxoma is a rare neoplasia with an obscure origin; its endothelial characteristics permit us to hypothesize that the factors inducing angiogenesis are also important for the growth and differentiation of this tumor type.

Cardiac tissue typically consists of mast cells in the myocardium and endocardium. Notably, such elements, as well as in cardiac myxomas, are predominantly located in the atrium (13).

Cell receptors and molecules of the extracellular matrix have previously been identified as possible factors of growth and angiogenesis in cardiac myxomas (14). However, previous literature has focused on the phenotype of cells, suggesting that cardiac myxoma cells may derive from adult developmental remnants in the presence of myocytic antigens (15). In such a setting, it is important to correlate our data with other available studies. CD117 expression may be considered as a key factor in order to distinguish putative cardiac progenitor cells (negative) from mast cells (positive) in child and adult human hearts (16).

Numerous observations support a model in which the endocardium is a spatially restricted population of the endothelium, arising as a result of *de novo* vasculogenesis from precursor cells present in the cardiac crescent (17). It is likely that myxoma cells are independent endocardial precursors of endothelial cells expressing CD31 (18).

The pattern of CD34 and CD31 expression and the reciprocal location of vascular and pseudovascular proliferation found in our series, suggests that in cardiac myxomas, endothelial precursors of endocardial type, through an intermediate stage of tumor cells, may also differentiate into vascular endothelia. CD34 is a cell surface glycoprotein expressed on hematopoietic stem and progenitor cells and on the luminal cell membrane of endothelial cells of small blood and lymphatic vessels (19–21). Moreover, a small subset of CD34-positive precursor cells remain present in later passages of primary endothelial cell cultures and also in immortalized endothelial cell lines (22). Such cells have been identified as endothelial elements able to regulate angiogenesis (23). In the present study, the pool of isolated elements of myxomas was CD34-positive. Such elements may be tumoral stem cells with angiogenic properties. Moreover, in our myxoma series, angiogenesis was often intermixed with pseudovascular structures. Thus, the angiogenic factors of mast cells may play a pivotal role in regulating the growth and differentiation of such a primitive endothelial population.

The ambiguous correlation between mast cells and tumors has been previously investigated (24,25) and among the functions of mast cells promoting tumor development, their contribution to neoangiogenesis appears to be highly important. Angiogenesis was measured and microvessels were counted in human endometrial carcinoma (26). The number of microvessels correlated with the number of tryptase-positive cells and these parameters increased with tumor progression. A similar outcome was observed in uterine cervix carcinoma (7), pulmonary adenocarcinoma (27) and gastrointestinal cancers (28). The density of mast cells is also parallel to microvessel density in the progression of gastric carcinoma (29). This correlation was observed for chymase- and tryptase-positive cells.

In conclusion, the present study demonstrated a significant correlation between angiogenesis and the number of mast cells present in cardiac myxomas. As tumors rich in pseudovascular structures contain a significantly higher number of mastocytes, such cellular elements may play a role in the development and differentiation of tumoral cells with an endothelial origin.

Finally, as tryptase is important for tumor progression, the inhibition of this proteinase is a promising technique in patients not surgically treatable. Compounds targeting tryptase, although designed as anti-allergenics, may also exert antitumor effects (25,30).

## Figures and Tables

**Table I:** Morphological patterns and immunohistochemical analysis of myxomas.

Case	Morphological patterns	No. of tryptase-/CD117-positive cells^a^	Blood vessel density score
1	APS, H	18.4/23.0	4
2	APS, H	12.6/14.5	4
3	APS	15.4/17.4	4
4	APS	7.6/11.4	3
5	SPS	6.2/6.6	3
6	SPS	6.0/7.4	2
7	SPS	8.2/10.4	2
8	SPS	8.8/12.4	3
9	SPS	5.4/7.0	1
10	SPS	6.4/9.2	1

APS, abundant pseudovascular structures; H, hemorrhagic; SPS, scanty pseudovascular structures; CD117, cluster of differentiation 117;

a Average calculated from five consecutive tissue slices of each case.

**Table II:** Tumor size (cm).

Case	Size
1	5.8
2	3.7
3	3.8
4	4.9
5	3.8
6	1.7
7	2.3
8	2.6
9	3.4
10	3.5
